# National Trends and Outcomes Associated With Presence and Type of Usual Clinician Among Older Adults With Multimorbidity

**DOI:** 10.1001/jamanetworkopen.2021.34798

**Published:** 2021-11-30

**Authors:** Ishani Ganguli, Claire McGlave, Meredith B. Rosenthal

**Affiliations:** 1 Harvard Medical School, Boston, Massachusetts; 2 Division of General Internal Medicine and Primary Care, Brigham and Women’s Hospital, Boston, Massachusetts; 3 Harvard T. H. Chan School of Public Health, Boston, Massachusetts

## Abstract

**Importance:**

Declining primary care visit rates and increasing specialist visit rates among older adults with multimorbidity raise questions about the presence, specialty, and outcomes associated with usual clinicians of care for these adults.

**Objective:**

To examine trends in the presence and specialty of usual clinicians and the association with preventive care receipt and spending.

**Design, Setting, and Participants:**

This survey study used repeated cross-sectional analyses of Medicare Current Beneficiary Survey data from 2010, 2013, and 2016. Participants were community-dwelling Medicare Advantage and traditional Medicare members with at least 2 chronic conditions. Data were analyzed from March 1, 2020, to February 5, 2021.

**Main Outcomes and Measures:**

Trends and factors associated with self-reported usual clinician presence and specialty. Multivariable regression was used to examine associations between usual clinician presence and specialty with preventive care receipt and spending, controlling for respondent sociodemographic and clinical characteristics.

**Results:**

A total of 25 490 unweighted respondent-years were examined, representing 90 324 639 respondent-years across the United States. Overall, 58.4% of respondent-years belonged to women, and the mean (SD) age of respondents was 77.5 (7.5) years. From 2010 to 2016, those reporting usual clinicians dropped from 94.2% to 91.0% (*P* < .001). Across study years, respondents were more likely to report a usual clinician if they were women (adjusted marginal difference [AMD], 2.5 percentage points; 95% CI, 1.5-3.5 percentage points) or had higher income (≥$50 000 vs <$15 000: AMD, 2.2 percentage points; 95% CI, 1.1-3.4 percentage points) and less likely if they were Black beneficiaries (vs White: AMD, −2.8 percentage points; 95% CI, −4.3 to −1.3 percentage points) or had traditional Medicare (vs Medicare Advantage: AMD, −3.2 percentage points; 95% CI. −4.1 to −2.3 percentage points). Among 23 279 respondents with usual clinicians, those reporting specialists as their usual clinicians decreased from 5.3% to 4.1% (*P* < .001). Across the study period, respondents were more likely to report specialists as their usual clinicians if they had traditional Medicare (vs Medicare Advantage: AMD, 2.3 percentage points; 95% CI, 1.6 to 2.9 percentage points), were Black or non-White Hispanic (Black vs White: AMD, 1.5 percentage points; 95% CI, 0.2 to 2.8 percentage points; non-White Hispanic vs White: AMD, 3.8 percentage points; 95% CI, 1.9 to 5.7 percentage points), or lived in the Northeast (vs Midwest: AMD, 3.6 percentage points; 95% CI, 2.1 to 5.2 percentage points). Compared with those without usual clinicians, respondents with usual clinicians were more likely to receive all examined preventive services, such as cholesterol screening (AMD, 6.7 percentage points; 95% CI, 5.4 to 8.1 percentage points) and influenza vaccines (AMD, 11.6 percentage points; 95% CI, 9.2 to 14.0 percentage points). Among respondents with usual clinicians, those reporting specialist usual clinicians (vs primary care) were less likely to receive influenza vaccines (AMD, −5.6 percentage points; 95% CI, −9.2 to −2.1).

**Conclusions and Relevance:**

In this study, older adults with multimorbidity were less likely to have a usual clinician over the study period, with potential implications for preventive care receipt. Our results suggest a key role for usual clinicians, especially primary care clinicians, in vaccination uptake for this population.

## Key Points

**Question:**

For older adults with multimorbidity, what are national trends in the presence and specialty of usual clinicians and their association with preventive care receipt and spending?

**Findings:**

In this survey study of 25 490 Medicare beneficiary–years, participants were less likely to report a usual clinician or have a specialist in this role over the study period. Those with usual clinicians were more likely to report preventive care receipt, while those with specialist usual clinicians reported lower influenza vaccination rates specifically.

**Meaning:**

In this study, older adults with multimorbidity were less likely to have a usual clinician over the study period, with implications for preventive care receipt.

## Teaser

This survey study examines trends in the presence and specialty of usual clinicians and their association with preventive care receipt and spending among older US adults with at least 2 chronic conditions.

## Introduction

For the growing population of older adults with multimorbidity (≥2 chronic conditions),^1^ having a usual clinician of care has been associated with better health outcomes, less high-acuity utilization, and lower costs.^2,3,4,5^ These usual clinicians can play a key role in providing whole-person continuous care, coordinating care across care settings and clinicians, and ensuring patients’ preventive care needs are met. Historically, usual clinicians have been primary care clinicians (PCPs) with specialties including family medicine and internal medicine.^2,3,6,7,8,9,10,11,12,13,14,15^ However, among older adults with multimorbidity, per capita PCP visits decreased by 20% in the past decade, while specialist visit rates increased.^16,17,18,19,20^ In light of these trends, it is unclear how access to a usual clinician has changed for older adults during this period and whether this care may be increasingly provided by specialist physicians.

In addition to having a usual clinician, the specialty of this clinician may have implications for care quality and utilization outcomes. For instance, having a specialist instead of a PCP as one’s usual clinician—as defined using office visit claims data—has been associated with greater spending and lower continuity among traditional Medicare (TM) beneficiaries, while another study found lower rates of emergency department visits and of potentially preventable hospitalizations among those with a specialist as their usual clinician.^21,22^ It remains unknown how usual clinician specialty relates to outcomes when considering patients’ self-reported usual clinicians or when including the now more than one-third of all Medicare beneficiaries enrolled in Medicare Advantage (MA), for whom network design may lead to greater reliance on primary care.^23^

Therefore, we used nationally representative survey data among older adults with multimorbidity enrolled in TM and MA to assess trends in self-report of having a usual clinician of care and of that clinician’s specialty. We then examined the association of these attributes with evidence-based preventive care receipt and spending.

## Methods

The Mass General Brigham institutional review board considered this study exempt from review and the requirement for informed consent per 45 CFR 46.101(b). The study follows the Strengthening the Reporting of Observational Studies in Epidemiology (STROBE) reporting guideline.

### Data Source

The Medicare Current Beneficiary Survey (MCBS) is a continuous in-person survey with rotating panel design of a representative national sample of TM and MA beneficiaries. The survey is conducted in English and Spanish and enriched for Hispanic beneficiaries and beneficiaries aged 85 years and older. MCBS collects self-reported health care utilization and cost data (including sources of payment for costs not covered by Medicare, such as copayments and deductibles). For TM beneficiaries, survey responses are linked to claims and administrative data using a matching algorithm. For MA beneficiaries, costs associated with survey-reported visits are reported using the MA explanation of benefits form. We linked the survey files and cost files from 2010, 2013, and 2016 using unique beneficiary identifiers (eFigure 1 in the Supplement).

### Study Population

In each study year, we examined TM and MA beneficiaries aged 65 years or older who were community-dwelling, continuously enrolled for that year, and had at least 2 self-reported chronic conditions collected in the survey, including hypertension, cardiovascular disease, pulmonary disease, arthritis, osteoporosis, diabetes, nonskin cancer, psychiatric disorders, Alzheimer disease or Alzheimer disease–related dementias (AD/ADRD), and Parkinson disease. We excluded beneficiaries residing in Puerto Rico due to differences in the Puerto Rico Medicare program (Puerto Rico has been removed from the MCBS sample since 2017).^24^

### Measures

#### Presence and Specialty of Usual Clinician

We assessed having a usual clinician of care based on affirmative responses to getting care in a usual place and seeing a usual doctor or other health professional there (survey language appears in eAppendix 1 in the Supplement). We categorized this usual clinician as a PCP based on patient-reported specialty in primary care (general practice, family practice, geriatric medicine, internal medicine, or osteopathy). We categorized the usual clinician as a specialist if they had any other specialty involving direct patient care in the outpatient setting (eg, cardiology) (all specialties are listed in eTable 1 in the Supplement). For analyses comparing respondents with primary care vs specialist usual clinicians, we excluded individuals who gave a response of other because we could not reliably assign them to either group.

#### Preventive Care

We assessed self-reported binary variables, including blood pressure checked in the past year, cholesterol checked in the past 5 years, diabetes screening ever received, colorectal cancer screening ever received, influenza vaccine received last winter, pneumonia vaccine ever received, and shingles vaccine ever received.

#### Spending

We assessed total and out-of-pocket spending using measures that summed payments from all payers for outpatient and inpatient costs (surgeon, physician, laboratory/imaging, durable medical equipment, and prescribed medicine costs) on an annual, per-beneficiary basis. Both spending measures used values imputed by MCBS for beneficiaries for whom full-year spending data were not collected. We standardized 2010 and 2013 spending to 2016 dollars using the Consumer Price Index for Medical Care.

#### Respondent Characteristics

We examined sex, age, race and ethnicity (using the Research Triangle Institute algorithm to improve identification of minority groups when available ^25,26^), income, educational status, marital status, living situation, primary insurance (MA vs TM), supplemental insurance (private, Medicaid, none), accountable care organization participation, geographic setting based on US Census Bureau’s urban-rural classification (metropolitan, ≥1 urbanized area with ≥50 000 inhabitants; micropolitan, ≥1 urban cluster with ≥10 000 inhabitants; or rural, population <10 000), census area, frailty quartile (using the validated MCBS-based frailty index^27^), number of chronic conditions (integer variable), and self-reported health. The racial and ethnic categories used in the survey were White, Black or African American, Hispanic, Asian, Native Hawaiian or Pacific Islander (2016 only), North American Native/American Indian or Alaska Native, do not know, other, more than 1 group, not ascertained, and refused to answer. We collapsed these groups into 4 categories: White, Black, non-White Hispanic, and other. These data were analyzed to examine race and ethnicity–based differences in care access.

### Statistical Analysis

We described rates of reporting a usual clinician of care and, among those who had a usual clinician, of reporting a specialist in this role. We presented these rates overall and by patient sociodemographic and clinical characteristic subgroups from 2010 to 2016. We used linear trend tests to determine the significance of overall trends in reporting a usual clinician and in the clinician’s specialty across the 3 survey years. We then assessed whether these trends varied significantly within member subgroups using models that included an interaction term between year and the given demographic or clinical categorical variable. Given that we used 3 years of data to assess trends, we also performed sensitivity analyses in which we repeated these analyses comparing 2010 to 2016 alone.

Then, to determine which characteristics were associated with having a usual clinician and that usual clinician being a specialist, we pooled data from 2010, 2013, and 2016 and built a series of multivariable logistic regression models including the previously mentioned demographic and clinical variables. We expressed results as adjusted marginal percentage point differences relative to the reference group.

#### Association With Preventive and Utilization Outcomes

To examine the association between usual clinician status and the previously described outcomes, we pooled 2010, 2013, and 2016 data after confirming that interactions between individual years and indicators for usual clinician status were nonsignificant. We compared outcomes among patients with or without a usual clinician using bivariable analyses, then created logistic regression models (for binary preventive care outcomes) and linear models (for spending outcomes), controlling for the previously mentioned covariates and presenting our results as adjusted marginal differences (AMDs). We repeated these steps to compare those with a PCP vs specialist as a usual clinician.

#### Specialist as Usual Clinician

Among patients with a specialist as a usual clinician, we described the frequency of various specialties playing that role in each year. We then examined the apparent alignment of specialists as usual clinicians of care with the patient’s care needs. Specifically, for each chronic condition captured in the survey, we determined which specialty or specialties traditionally included that condition within their area of focus (eTable 2 in the Supplement). Then, we estimated the mean and median proportion of beneficiaries’ chronic conditions that fell under the focus of their usual clinician’s specialty.

Throughout, we used MCBS cross-sectional survey weights that accounted for the stratified sampling design, survey nonresponse, coverage error, and repeated observations of beneficiaries across time to compute nationally representative estimates. We used Stata version 16.0 (StataCorp) and considered 2-tailed *P* values significant at *P* < .05. Missingness was addressed using complete case analysis (eAppendix 2 in the Supplement). Data were analyzed from March 1, 2020, to February 5, 2021.

## Results

We examined 25 490 unweighted respondent-years, representing 90 324 639 respondent-years. Overall, 58.4% of respondent-years belonged to women, and the mean (SD) age of respondents was 77.5 (7.5) years. From 2010 to 2016, the percentage of all older adults with multimorbidity who reported a usual clinician dropped from 94.2% to 91.0% overall (−3.2 percentage points; *P* < .001 for trend) (Table 1). This result was observed for nearly all demographic or clinical subgroups. Older adults in the Northeast were slightly more likely to have a usual clinician, while other regions saw a significant decline. In addition, non-White Hispanic adults and those of other race or ethnicity saw an increase in having a usual clinician, while White and Black adults saw a decrease. There were no other significant differences in this trend within groups. In the sensitivity analyses comparing 2010 to 2016 proportions, our results were identical (eTable 3 in the Supplement).

**Table 1.** Trends and Patient- and Area-Level Characteristics Associated With Reporting a Usual Clinician of Care^a^

Characteristic	Respondents, No. (%)^b^		Difference 2010-2016, percentage points	*P* value for trend	Adjusted marginal difference between subgroups, percentage point (95% CI)^c^
	2010 (n = 8677)	2016 (n = 8017)			
Overall	8178 (94.2)	7332 (91.0)	−3.2	<.001	NA
Sex					
Male	3304. (93.6)	3061 (90.3)	−3.3	.72	[Reference]
Female	4874 (94.6)	4271 (91.5)	−3.1		2.5 (1.5 to 3.5)
Age, y					
65-74	3178 (94.0)	2625 (90.6)	−3.4	.90	[Reference]
75-84	3468 (94.9)	3071 (92.1)	−2.8		1.6 (0.7 to 2.5)
≥85	1532 (93.4)	1636 (90.2)	−3.2		1.2 (0.1 to 2.3)
Income, $^d^					
<15 000	1809 (91.0)	1386 (86.1)	−4.9	.67	[Reference]
15 000-29 999	2087 (93.9)	1991 (90.0)	−3.9		0.7 (−0.5 to 1.9)
30 000-49 999	1766 (95.5)	1500 (92.5)	−3.0		1.6 (0.2 to 3.0)
≥50 000	1417 (96.9)	2455 (93.2)	−3.7		2.2 (1.1 to 3.4)
Race and ethnicity					
White	6577 (95.2)	5889 (91.5)	−3.7	.01	[Reference]
Black	698 (91.1)	628 (86.3)	−4.8		−2.8 (−4.3 to −1.3)
Non-White Hispanic	513 (89.5)	562 (91.7)	2.2		−0.7 (−2.3 to 1.0)
Other^e^	367 (90.7)	253 (91.3)	0.6		0.3 (−1.2 to 1.9)
Education					
Did not complete high school	2048 (91.7)	1428 (87.2)	−4.6	.99	[Reference]
High school or above	6130 (95.0)	5904 (91.8)	−3.2		1.8 (0.7 to 2.9)
Marital status					
Not married	3978 (92.9)	3560 (88.5)	−4.3	.81	[Reference]
Married	4200 (95.4)	3772 (93.0)	−2.4		3.0 (1.8 to 4.1)
Living situation					
Lives alone	2675 (93.4)	2475 (89.5)	−4.0	.81	−0.4 (−1.3 to 0.6)
Lives with others	5503 (94.6)	4857 (91.7)	−2.9		[Reference]
Primary insurance					
TM	5991 (94.1)	5445 (90.5)	−3.6	.35	−3.2 (−4.1 to −2.3)
MA	2187 (94.5)	1887 (92.4)	−2.1		[Reference]
Supplemental insurance					
Medicaid	933 (89.5)	985 (86.0)	−3.5	.81	0.6 (−1.1 to 2.4)
Commercial	4653 (95.8)	3882 (93.2)	−2.6		3.7 (2.7 to 4.8)
None	2592 (93.1)	2465 (89.8)	−3.3		[Reference]
ACO participation					
Enrolled in an ACO	NA	1658 (93.6)	NA	NA	NA
Not enrolled in an ACO		5674 (90.4)			
Rural-urban status					
Metropolitan	NA	5600 (8.5)	NA	NA	NA
Micropolitan		1101 (87.7)			
Rural		631 (91.1)			
Census area					
Northeast	1392 (94.2)	1332 (94.5)	0.2	<.001	1.0 (−0.2 to 2.1)
Midwest	1974 (95.0)	1866 (91.7)	−3.4		[Reference]
South	3342 (95.2)	2797 (90.3)	−4.9		−0.0 (−1.2 to 1.2)
West	1457 (91.4)	1337 (88.4)	−3.0		−3.1 (−4.6 to −1.6)
Frailty quartiles					
First	1989 (94.7)	1890 (91.8)	−2.9	.53	[Reference]
Second	2095 (94.9)	1829 (91.6)	−3.3		−0.3 (−1.5 to 1.0)
Third	2078 (94.3)	1785 (89.9)	−4.4		−1.2 (−2.4 to −0.1)
Fourth	2016 (92.8)	1828 (90.3)	−2.5		−1.0 (−2.5 to 0.6)
Chronic conditions, No.					
2	2563 (93.8)	2250 (90.1)	−3.7	.74	[Reference]
3	2478 (94.1)	2185 (91.1)	−3		1.5 (0.6 to 2.5)
4	1715 (95.7)	1559 (92.3)	−3.3		2.4 (1.2 to 3.6)
≥5	1422 (94.6)	1338 (91.0)	−3.7		2.7 (1.4 to 4.0)
Self-reported health					
Excellent, very good, or good	6194 (94.8)	5722 (91.4)	−3.3	.44	1.2 (0.1 to 2.2)
Fair or poor	1964 (93.2)	1610 (89.5)	−3.7		[Reference]

Abbreviations: ACO, accountable care organization; MA, Medicare Advantage; NA, not applicable; TM, traditional Medicare.

^a^ Data source was Centers for Medicare & Medicaid Services, Medicare Current Beneficiary Survey, Survey File Data, 2010, 2013, 2016.

^b^ Overall, 25 490 respondent-years were included in this analysis. All percentages are weighted.

^c^ The multivariable model used pooled data from 2010, 2013, and 2016 and included all variables presented in the table other than ACO participation and rural-urban status (only asked in 2016). This multivariable model excluded 93 respondents for whom 1 or more responses were missing.

^d^ Income reported as do not know or refused to answer was treated as a separate category.

^e^ Other includes participants who selected Asian, Native Hawaiian or Pacific Islander (2016 only), North American Native/American Indian or Alaska Native, do not know, other, more than 1 group, not ascertained, and refused to answer.

Across the study period, adults were more likely to report having a usual clinician if they were female, were older, had higher income, had a high school or greater education, were married, had commercial supplemental insurance (vs none), and had more chronic conditions. Adults were less likely to have a usual clinician if they were Black (vs White) or if they had TM (vs MA).

Among 23 625 adults with a usual clinician, 23 279 had a clinician type that could be categorized as primary care vs specialist. Those with a specialist in this role declined from 5.3% to 4.1% (−1.2 percentage points; *P* < .001 for trend) (Table 2). This decrease was also present in most demographic and clinical subgroups, and there were no significant differences within groups. These findings were robust to our sensitivity analyses comparing 2010 to 2016 proportions (eTable 4 in the Supplement).

**Table 2.** Trends and Patient and Area-Level Characteristics Associated With Reporting a Specialist as the Usual Clinician of Care^a^

Characteristic	Respondents, No. (%)^b^		Difference 2010-2016, percentage points	*P* value for trend	Adjusted marginal difference between subgroups, percentage points (95% CI)^c^
	2010 (n = 8116)	2016 (n = 7158)			
Overall	428 (5.3)	272 (4.1)	−1.2	<.001	NA
Sex					
Male	178 (5.5)	110 (4.2)	−1.3	.55	[Reference]
Female	250 (5.2)	162 (4.0)	−1.2		−0.7 (−1.5 to 0.1)
Age, y					
65-74	171 (5.3)	97 (4.1)	−1.3	.78	[Reference]
75-84	171 (5.1)	104 (3.8)	−1.3		0.0 (−0.6 to 0.6)
≥85	86 (5.9)	71 (4.8)	−1.1		0.3 (−0.6 to 1.2)
Income, $^d^					
<15 000	93 (5.0)	50 (3.9)	−1.1	.30	[Reference]
15 000-29 999	101 (5.0)	76 (4.5)	−0.5		−0.5 (−1.4, 0.3)
30 000-49 999	92 (5.4)	44 (3.1)	−2.2		−1.0 (−2.0 to 0.1)
≥50 000	91 (6.2)	102 (4.5)	−1.7		0.1 (−1.2 to 1.3)
Race and ethnicity					
White	334 (5.1)	200 (3.7)	−1.4	.40	[Reference]
Black	41 (6.5)	30 (5.8)	−0.7		1.5 (0.2 to 2.8)
Non-White Hispanic	37 (7.2)	30 (5.6)	−1.7		3.8 (1.9 to 5.7)
Other^e^	14 (3.7)	12 (5.1)	1.4		0.6 (−1.2 to 2.3)
Education					
Did not complete high school	91 (4.5)	46 (3.4)	−1.1	.83	[Reference]
High school or greater	337 (5.6)	226 (4.3)	−1.3		1.4 (0.6 to 2.3)
Marital status					
Not married	220 (5.5)	146 (4.7)	−0.8	.39	[Reference]
Married	208 (5.2)	126 (3.6)	−1.6		−0.9 (−2.1 to 0.2)
Living situation					
Lives alone	151 (5.7)	100 (4.9)	−0.8	.89	0.4 (−0.6 to 1.3)
Lives with others	277 (5.2)	172 (3.8)	−1.4		[Reference]
Primary insurance					
TM	353 (6.1)	226 (4.7)	−1.4	.99	2.3 (1.6 to 2.9)
MA	75 (3.3)	46 (2.5)	−0.8		[Reference]
Supplemental insurance					
Medicaid	38 (4.1)	35 (3.6)	−0.6	.55	−1.3 (−2.2 to −0.3)
Commercial	272 (6.1)	155 (4.4)	−1.7		0.4 (−0.5 to 1.3)
None	118 (4.4)	82 (3.8)	−0.6		[Reference]
ACO participation					
Enrolled in an ACO	NA	73 (5.0)	NA	NA	NA
Not enrolled in an ACO		199 (3.9)			
Rural-urban status					
Metropolitan	NA	234 (4.5)	NA	NA	NA
Micropolitan		28 (2.5)			
Rural		10 (1.8)			
Census area					
Northeast	86 (6.5)	75 (7.4)	0.9	.23	3.6 (2.1 to 5.2)
Midwest	81 (4.3)	45 (2.8)	−1.5		[Reference]
South	182 (5.5)	100 (3.4)	−2.2		0.7 (−0.2 to 1.5)
West	78 (5.0)	52 (3.8)	−1.2		0.4 (−0.5 to 1.2)
Frailty quartiles					
First	79 (3.9)	64 (3.6)	−0.3	.47	[Reference]
Second	109 (5.6)	60 (4.0)	−1.6		0.8 (0.0 to 1.6)
Third	117 (5.6)	65 (4.5)	−1.1		1.1 (0.2 to 2.1)
Fourth	123 (6.4)	83 (4.6)	−1.9		1.6 (0.5 to 2.8)
Chronic conditions, No.					
2	125 (5.2)	86 (4.5)	−0.7	.46	[Reference]
3	127 (5.0)	88 (4.3)	−0.8		−0.3 (−1.1 to 0.6)
4	89 (5.2)	50 (3.6)	−1.5		−0.8 (−1.8 to 0.2)
≥5	87 (6.3)	48 (3.5)	−2.7		−0.7 (−1.7 to 0.3)
Self-reported health					
Excellent, very good, or good	307 (4.9)	202 (4.0)	−0.9	.99	−0.8 (−1.7 to 0.1)
Fair or poor	120 (6.7)	70 (4.5)	−2.2		[Reference]

Abbreviations: ACO, accountable care organization; MA, Medicare Advantage; NA, not applicable; TM, Traditional Medicare.

^a^ Data source was Centers for Medicare & Medicaid Services, Medicare Current Beneficiary Survey, Survey File Data, 2010, 2013, 2016.

^b^ Overall, 23 297 respondent-years were included in this analysis. All percentages are weighted.

^c^ The multivariable model used pooled data from 2010, 2013, and 2016 and included all variables presented in the table other than ACO participation and rural-urban status (only asked in 2016). This multivariable model excluded 75 respondents for whom 1 or more responses were missing.

^d^ Income reported as do not know or refused to answer was treated as a separate category.

^e^ Other includes participants who selected Asian, Native Hawaiian or Pacific Islander (2016 only), North American Native/American Indian or Alaska Native, do not know, other, more than 1 group, not ascertained, and refused to answer.

Across the study period, 22 242 respondent-years reported a PCP and 1037 reported a specialist in this role. Adults were more likely to report a specialist as their usual clinician if they had TM (vs MA: AMD, 2.3 percentage points; 95% CI, 1.6 to 2.9 percentage points), were Black or non-White Hispanic (Black vs White: AMD, 1.5 percentage points; 95% CI, 0.2 to 2.8 percentage points; Hispanic vs White: AMD, 3.8 percentage points; 95% CI, 1.9 to 5.7 percentage points), lived in the Northeast (vs Midwest: AMD, 3.6 percentage points; 95% CI, 2.1 to 5.2 percentage points), had a high school education or greater (vs did not complete high school: AMD, 1.4 percentage points; 95% CI, 0.6 to 2.3 percentage points), and had frailty (top quartile vs bottom quartile in frailty index: AMD, 1.6 percentage points; 95% CI, 0.5 to 2.8 percentage points). Those with supplementary Medicaid coverage were less likely to have a specialist (vs no supplemental insurance: AMD, −1.3 percentage points; 95% CI, −2.2 to −0.3 percentage points).

### Outcomes

In bivariable analyses, adults with a usual clinician were more likely to receive all examined preventive services and had higher total and out-of-pocket spending (Table 3). When adjusting for patient and area-level covariates, adults with a usual clinician remained more likely to report receipt of all examined services. They were more likely to report having been screened for hypertension (AMD, 6.3 percentage points; 95% CI, 4.6-7.9 percentage points), hyperlipidemia (AMD, 6.7 percentage points; 95% CI, 5.4-8.1 percentage points), diabetes (AMD, 4.3 percentage points; 95% CI, 1.2-7.4 percentage points), and colorectal cancer (AMD, 4.6 percentage points; 95% CI, 1.4-7.9 percentage points). They were also more likely to report having received their seasonal influenza vaccine (AMD, 11.6 percentage points; 95% CI, 9.2-14.0 percentage points), pneumonia vaccine (AMD, 6.5 percentage points; 95% CI, 3.8-9.2 percentage points), and shingles vaccine (AMD, 6.3 percentage points; 95% CI, 2.2-10.5 percentage points). Having a usual clinician was also associated with higher total spending (AMD, $891.75; 95% CI, $15.61-$1767.90).

**Table 3.** Quality and Utilization Outcomes Associated With Reporting a Usual Clinician^a^

Outcome	Respondents, No. (%)^b^		Unadjusted difference, percentage point	*P* value for unadjusted difference	Adjusted marginal difference, percentage point (95% CI)^c^
	No usual clinician (n = 1865)	Usual clinician (n = 23 625)			
**Receipt of preventive care**					
Blood pressure checked	1717 (91.2)	23 363 (98.8)	7.6	<.001	6.3 (4.6 to 7.9)
Cholesterol checked	1639 (88.2)	22 762 (96.8)	8.5	<.001	6.7 (5.4 to 8.1)
Diabetes screening	556 (46.3)	8375 (53.0)	6.7	.006	4.3 (1.2 to 7.4)
Colorectal cancer screening	452 (34)	5729 (41.2)	7.2	<.001	4.6 (1.4 to 7.9)
Influenza vaccine	1130 (57.6)	17 681 (73.4)	15.9	<.001	11.6 (9.2 to 14.0)
Pneumonia vaccine	1258 (64)	18 147 (74.7)	10.7	<.001	6.5 (3.8 to 9.2)
Shingles vaccine	180 (26.2)	2675 (36.9)	10.8	.02	6.3 (2.2 to 10.5)
**Spending**					
**Outcome**	**No usual clinician, mean (SD), (n = 1755), $**	**Usual clinician, mean (SD), (n = 21 360), $**	**Difference, mean, $**	***P *value for difference**	**Mean (95% CI), $**
Total spending	5066.24 (283.55)	6592.75 (95.38)	1526.51	<.001	891.75 (15.61 to 1767.90)
Out-of-pocket spending	708.96 (61.19)	878.59 (28.56)	169.63	.02	31.87 (−158.49 to 222.23)

^a^ Data source was Centers for Medicare & Medicaid Services, Medicare Current Beneficiary Survey, Survey File Data, 2010, 2013, 2016. Diabetes screening asked in 2010 and 2016 only; colorectal cancer screening asked in 2013 and 2016 only; shingles vaccine question asked in 2016 only.

^b^ Overall, 25 490 respondent-years were included in this analysis. All percentages are weighted.

^c^ The multivariable models for preventive care adjusted for sex, age, race and ethnicity, income, educational status, marital status, living situation, primary insurance, supplemental insurance, census area, frailty quartile, number of chronic conditions, and self-reported health and excluded 93 respondents with 1 or more missing responses. Income reported as do not know or refused to answer was treated as a separate category. The multivariable models for cost adjusted for the same covariates and excluded 2375 respondents (9%) with missing responses.

In bivariable analyses among adults with a usual clinician, those with a specialist clinician were less likely to have gotten their influenza vaccine and had higher spending (Table 4). When adjusting for covariates, those with a specialist clinician remained less likely to have gotten their influenza vaccine (AMD, −5.6 percentage points; 95% CI, −9.2 to −2.1 percentage points) but there were no significant differences in receipt of other preventive services or in spending.

**Table 4.** Quality and Utilization Outcomes Associated With Having a Specialist as Usual Clinician^a^

Outcome	Respondents, No. (%)^b^		Unadjusted difference, percentage point	*P* value for unadjusted difference	Adjusted marginal difference, percentage point (95% CI)^c^
	PCP (n = 22 242)	Specialist (n = 1037)			
**Receipt of preventive care**					
Blood pressure checked	21 995 (98.8)	1029 (99.3)	0.4	.27	0.4 (−0.2 to 1.0)
Cholesterol checked	21 443 (96.8)	994 (96.3)	−0.5	.50	−0.4 (−1.9 to 1.1)
Diabetes screen	7851 (52.8)	388 (54.7)	1.9	.60	2.3 (−1.9 to 6.6)
Colorectal cancer screening	5396 (41.2)	199 (37.3)	−3.8	.08	−4.7 (−9.6 to 0.1)
Influenza vaccine	16 721 (73.8)	728 (68)	−5.8	.001	−5.6 (−9.2 to −2.1)
Pneumonia vaccine	17 119 (74.9)	777 (72.6)	−2.3	.14	−1.9 (−5.1 to 1.3)
Shingles vaccine	2542 (37.3)	79 (30.2)	−7.1	.01	−5.9 (−12.5 to 0.6)
**Spending**					
**Outcome **	**PCP, mean (SD), $ (n = 20 249)**	**Specialist, mean (SD), $ (n = 954)**	**Unadjusted difference, mean, $**	***P* value for unadjusted difference**	**Adjusted marginal difference, mean (95% CI), $**
Total spending	6524.46 (96.01)	8608.18 (624.3)	2083.7	.003	1042.75 (−73.27 to 2158.77)
Out-of-pocket spending	870.93 (29.03)	1060.67 (131.28)	189.74	.19	−33.08 (−240.46 to 174.30)

Abbreviation: PCP, primary care clinician.

^a^ Data source was Centers for Medicare & Medicaid Services, Medicare Current Beneficiary Survey, Survey File Data, 2010, 2013, 2016. Diabetes screening asked in 2010 and 2016 only; colorectal cancer screening asked in 2013 and 2016 only; shingles vaccine question asked in 2016 only.

^b^ Overall, 23 279 respondent-years were included in this analysis. All percentages are weighted.

^c^ The multivariable models for preventive care adjusted for sex, age, race/ethnicity, income, educational status, marital status, living situation, primary insurance, supplemental insurance, census area, frailty quartile, number of chronic conditions, and self-reported health and excluded 75 respondents with 1 or more missing responses. Income reported as do not know or refused to answer was treated as a separate category. The multivariable models for cost adjusted for the same covariates and excluded 2076 respondents (9%) with missing responses.

Beneficiaries with a specialist usual clinician most often reported that their specialty was cardiology (35.2% of beneficiaries with specialist usual clinicians) followed by endocrinology (14.7%) (eFigure 2 in the Supplement). Among beneficiaries with a specialist usual clinician, a mean (SD) of 24% (25%) and a median (IQR) of 25% (0-43%) of their chronic conditions fell under that specialty’s area of focus.

## Discussion

In this nationally representative multiyear analysis, older adults with multimorbidity enrolled in TM and MA were less likely over time to report a usual clinician and to have a specialist in that role. Respondents with a usual clinician were more likely to report receiving all examined preventive care measures, while reporting a specialist usual clinician was associated with lower influenza vaccination rates.

Fewer older adults with multimorbidity reported a usual clinician from 2010 to 2016, raising concerns for this high-need population. This result mirrors another survey study showing a decrease between 2002 and 2015 in having primary care (defined by primary care functions) among adults in their 70s with at least 3 conditions.^28^ A variety of factors may explain the decrease we observed, such as increasing financial barriers to accessing care,^19^ changing perceptions about patient-clinician relationships, decreased availability of PCPs,^29^ and care models that increasingly prioritize access over interpersonal continuity of care.^30^

We found that most adults in our study continued to have PCPs as usual clinicians, consistent with prior research on adults with serious illness and multiple chronic conditions having a generalist usual clinician.^31^ This finding, in light of well-established declines in primary care visit rates, may be consistent with evidence of PCPs and teams delivering longer and more comprehensive (if less frequent) visits and providing care outside of the context of visits, eg, through portal messages and telephone calls.^17,20^ In parallel, there was a small decrease in having a specialist as a usual clinician, such that the established increase in specialist visit rates may not be explained by more specialist usual clinicians.^16^ This raises the possibility that these additional visits may contribute to care fragmentation.^2,21,32,33,34,35,36,37,38,39^ As of 2000, fee-for-service Medicare beneficiaries saw a median of 2 PCPs and 5 specialists, and the latter number has likely increased.^40^

Racial and socioeconomic differences in reporting a usual clinician, even after accounting for insurance coverage, were unfortunately consistent with broader literature demonstrating worse access to care for those in racial/ethnic minority and lower socioeconomic status groups.^19,41^ These disparities may reflect geographic barriers in light of primary care workforce maldistribution and health care facilities moving out of racial and ethnic minority and lower-income areas,^42^ digital access barriers,^43,44^ and interpersonal, institutional, and structural racism.^45,46^ Indeed, Black individuals have been shown to have lower than average use of any ambulatory care yet greater than average use of emergency department and inpatient care, highlighting missed opportunities to prevent poor health outcomes.^47^

More encouragingly, enrollment in MA, which is increasing,^48^ was associated with reporting a usual clinician and having that clinician be a PCP. These results are consistent with MA beneficiaries using more primary care services and having network designs promoting greater reliance on primary care.^23,49^ Of note, a Health and Retirement Study analysis found no difference in having a usual clinician between TM and MA enrollees, possibly because they focused on higher-need beneficiaries.^50^

When considering outcomes, reporting a usual clinician was associated with substantially higher rates of all examined screening and preventive care measures. These findings build on a study by Blewett et al,^5^ which showed that having a usual clinician was associated with more screening/preventive tests among those aged 50 to 64 years.^5^

When we examined differences in outcomes among patients with a specialist vs primary care usual clinician, we found that those with specialist usual clinicians had similar outcomes (eg, rates of blood pressure, cholesterol, and diabetes screening), reflecting the important role of specialists in the care of older adults.^51^ However, those with specialist usual clinicians had lower vaccination rates, with differences in influenza vaccination rates reaching statistical significance. This likely reflects not only trusting relationships between PCPs and patients but also primary care infrastructure and workflows designed to ensure delivery of these services in clinic or to remind patients to get these vaccines at local pharmacies.

Those with specialist usual clinicians had higher costs in bivariable analyses, but these differences did not reach statistical significance when accounting for patient risk factors. This is in contrast to prior work showing higher costs with specialist usual clinicians; those studies based the definition of usual clinician on claims-based visit ratios rather than patient self-report.^21,52,53^ Patient self-report is more closely aligned with patient experience, reflects nonvisit interactions, and avoids issues of circularity (a specialist is attributed as one’s usual clinician based on frequency of visits and may increase spending via these visits); however, visit-based attribution may better capture de facto usual care even if patients do not perceive it as such. It may also be that patients who view specialists as their usual clinicians prioritize care for a single condition and seek less routine care overall, helping to explain the lack of spending differences despite other evidence that primary care use reduces costs.^2^

Our results suggest the importance of providing usual care—ideally primary care—for older adults with multimorbidity. In addition to the preventive health benefits described previously in this study, specialist usual clinicians had primary focus in just 24% to 25% of their patients’ chronic conditions, suggesting potential gaps in care that were not captured by the outcomes we examined. In parallel, it will be important to ensure that specialists who serve as usual clinicians can offer routine vaccinations through the use of technology (eg, electronic health record prompts) and partnerships with pharmacies.

### Limitations

This study should be interpreted in the context of its limitations. While we view the self-reported usual source of care measure as a strength of the study, this measure is subject to potential recall errors and differences in interpretation. Survey limitations did not allow us to distinguish between clinician types (eg, physician vs nurse practitioner). Although we controlled outcome models for multiple observable sociodemographic and clinical variables, this study was not designed to infer causality. Five of the preventive care outcomes measured having received the service in the past 5 years or ever, which may bias the association of these outcomes with current presence of usual clinician to the null. Additionally, we did not examine differences between subspecialties in provision of preventive care.

## Conclusions

Access to usual care, and especially primary care, may have preventive health benefits for older adults with multimorbidity. The decrease in usual care access that we observed from 2010 through 2016 may have persisted and even accelerated to date, given COVID-19 pandemic–era financial struggles and likely closures among primary care practices.^54,55^ Bolstering equitable access to usual care, and especially primary care, requires a multipronged approach, including primary care workforce expansion and redistribution to areas of need, insurance design to encourage regular primary care engagement, and technology-enabled efficiencies in care delivery.^16,19^ In the shorter term, to the extent our results suggest primary care relationships may promote vaccine uptake specifically, health care leaders and policy makers should enable PCPs to leverage these relationships for public health benefit during the COVID-19 pandemic and beyond.^56^

## Notes

Supplement. **eFigure 1. **Data Structure **eAppendix 1. **Survey Questions Used for Usual Clinician Definitions **eTable 1. **Definitions of Primary Care and Specialty Clinicians **eTable 2. **Chronic Conditions and Relevant Specialty Types **eAppendix 2. **Missingness **eTable 3. **Trends and Patient and Area-Level Characteristics Associated With Reporting a Usual Clinician of Care, Additional Details **eTable 4.** Trends and Patient and Area-Level Characteristics Associated With Reporting a Specialist as the Usual Clinician of Care, Additional Details **eFigure 2. **Specialties of Usual Clinicians Among Respondents Reporting a Specialist in This Role, 2010-2016
