# Supplementary material for: National Trends and Outcomes Associated With Presence and Type of Usual Clinician Among Older Adults With Multimorbidity
Source: JAMA Netw Open. 2021 Nov 30;4(11):e2134798. doi: 10.1001/jamanetworkopen.2021.34798 (PMC8634053; doi:10.1001/jamanetworkopen.2021.34798)
Supplement: Supplement. — eFigure 1. Data Structure eAppendix 1. Survey Questions Used for Usual Clinician Definitions eTable 1. Definitions of Primary Care and Specialty Clinicians eTable 2. Chronic Conditions and Relevant Specialty Types eAppendix 2. Missingness eTable 3. Trends and Patient and Area-Level Characteristics Associated With Reporting a Usual Clinician of Care, Additional Details eTable 4. Trends and Patient and Area-Level Characteristics Associated With Reporting a Specialist as the Usual Clinician of Care, Additional Details eFigure 2. Specialties of Usual Clinicians Among Respondents Reporting a Specialist in This Role, 2010-2016 [file jamanetwopen-e2134798-s001.pdf]

Supplemental Online Content

Ganguli I, McGlave C, Rosenthal MB. National trends and outcomes associated with presence and type of usual clinician among older adults with multimorbidity. *JAMA Netw Open.* 2021;4(11):e2134798. doi:10.1001/jamanetworkopen.2021.34798

eFigure 1. Data Structure

eAppendix 1. Survey Questions Used for Usual Clinician Definitions

eTable 1. Definitions of Primary Care and Specialty Clinicians

eTable 2. Chronic Conditions and Relevant Specialty Types

eAppendix 2. Missingness

eTable 3. Trends and Patient and Area-Level Characteristics Associated With Reporting a Usual Clinician of Care, Additional Details

eTable 4. Trends and Patient and Area-Level Characteristics Associated With Reporting a Specialist as the Usual Clinician of Care, Additional Details

eFigure 2. Specialties of Usual Clinicians Among Respondents Reporting a Specialist in This Role, 2010-2016

This supplemental material has been provided by the authors to give readers additional information about their work.

© 2021 Ganguli I et al. *JAMA Network Open.*

eFigure 1. Data Structure

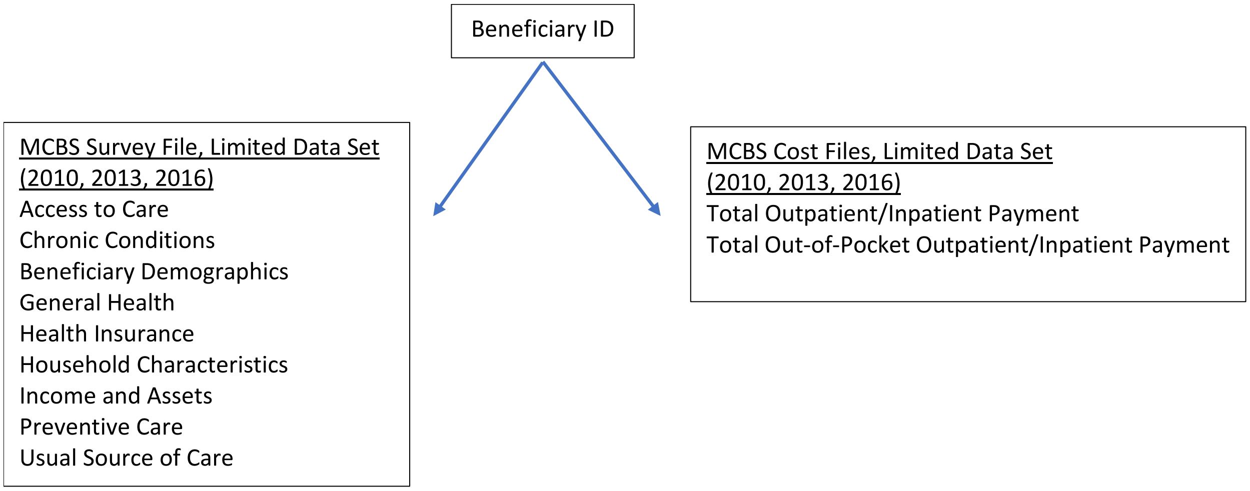

© 2021 Ganguli I et al. *JAMA Network Open.*

eAppendix 1. Survey Questions Used for Usual Clinician Definitions

2010 + 2013 – Selected Survey Questions from Community-Based Survey

1. Is there a particular medical person or a clinic you usually go to when you are sick or for advice about your health?

2. What kind of place do you usually go to when you are sick or for advice about your health -- is that a managed care plan or HMO center, a clinic, a doctor's office, a hospital, or some other place?

3. Is there a particular doctor you usually see at this [place/managed care plan/HMO center]?

4. What is [Provider Name's] specialty?

2016 – Selected Survey Questions from Community-Based Survey

1. Is there a particular doctor or other health professional, or a clinic you usually go to when you are sick or for advice about your health?

2. What kind of place do you usually go to when you are sick or for advice about your health -- is that a managed care plan or HMO center, a clinic, a doctor's office, a hospital, or some other place?

3. Is there a particular doctor or other health professional you usually see at this [place/managed care plan/HMO center]?

4. What is [Provider Name's] specialty?

© 2021 Ganguli I et al. *JAMA Network Open.*

eTable 1. Definitions of Primary Care and Specialty Clinicians

Role	Specialties
Primary care	Family Practice<br>General Practice<br>Geriatrics<br>Hospital Residence<br>Internal Medicine<br>Osteopathy
Specialty	Allergy/Immunology<br>Anesthesiology<br>Cardiology<br>Dermatology<br>Endocrinology<br>Gastroenterology<br>General Surgery<br>Ob/Gyn<br>Hematology<br>Nephrology<br>Neurology<br>Oncology<br>Ophthalmology<br>Orthopedic Surgery<br>Otorhinolaryngology<br>Physical Medicine and Rehabilitation<br>Plastic Surgery<br>Proctology<br>Psychiatry<br>Pulmonology<br>Rheumatology<br>Thoracic Surgery<br>Urology

© 2021 Ganguli I et al. *JAMA Network Open.*

eTable 2. Chronic Conditions and Relevant Specialty Types

Chronic Condition	Relevant Specialty
Hypertension	Cardiology
Cardiovascular disease including hardening of arteries, angina/coronary heart disease, myocardial infarction	Cardiology
Congestive heart failure	Cardiology
Other heart condition	Cardiology
Pulmonary disease	Pulmonology
Rheumatoid Arthritis	Rheumatology
Osteoarthritis / Arthritis (other)	Orthopedics or Rheumatology
Osteoporosis	Endocrinology
Diabetes	Endocrinology
Stroke	Neurology
Alzheimer’s Disease or Alzheimer’s Disease-related dementias	Neurology
Parkinson's disease	Neurology
Depression	Psychiatry
Mental or psychiatric disorder	Psychiatry

© 2021 Ganguli I et al. *JAMA Network Open.*

eAppendix 2. Missingness

Across 25,490 respondent-years, race was missing for 63 respondent-years (0.25%), Census area was missing for 21 respondent-years (0.08%), and self-reported health was missing for 80 respondent-years (0.31%). Total and out-of-pocket spending were missing for 2,375 respondent-years (9.3%).

eTable 3. Trends and Patient and Area-Level Characteristics Associated With Reporting a Usual Clinician of Care, Additional Details

		2010	2013	2016	Difference 2010 – 2016	P value for trend test	P value for chi square, 2010 vs 2016	Adjusted marginal percentage point difference between subgroups
		No. (%) N = 8,677	No. (%) N = 8,796	No. (%) N = 8,017	% point			% point (95% CI) N = 25,397
**Overall**		**8,178 (94.2)**	**8,115 (91.6)**	**7,332 (91.0)**	-3.2	**<.001**	**<.001**	**--**
Sex	Male	3,304. (93.6)	3,397 (90.4)	3,061 (90.3)	-3.3	0.72	0.77	Ref
	Female	4,874 (94.6)	4,718 (92.4)	4,271 (91.5)	-3.1			2.5 (1.5, 3.5)
Age	65-74	3,178 (94.0)	3,248 (90.8)	2,625 (90.6)	-3.4	0.90	0.95	Ref
	75-84	3,468 (94.9)	3,403 (92.7)	3,071 (92.1)	-2.8			1.6 (0.7, 2.5)
	85+	1,532 (93.4)	1,464 (92.6)	1,636 (90.2)	-3.2			1.2 (0.1 2.3)
Income	<$15,000	1,809 (91.0)	1,510 (87.0)	1,386 (86.1)	-4.9	0.67	0.70	Ref
	$15,000-$29,999	2,087 (93.9)	2,211 (91.2)	1,991 (90.0)	-3.9			0.7 (-0.5, 1.9)
	$30,000-$49,999	1,766 (95.5)	1,784 (93.0)	1,500 (92.5)	-3.0			1.6 (0.2, 3.0)
	$50,000+	1,417 (96.9)	1,903 (94.4)	2,455 (93.2)	-3.7			2.2 (1.1, 3.4)

© 2021 Ganguli I et al. *JAMA Network Open.*

Race	White	6,577 (95.2)	6,435 (92.5)	5,889 (91.5)	-3.7	0.010	0.012	Ref
	Black	698 (91.1)	717 (87.4)	628 (86.3)	-4.8			-2.8 (-4.3, -1.3)
	Asian/Native/Unknown/Other	367 (90.7)	385 (91.8)	253 (91.3)	0.6			0.3 (-1.2, 1.9)
	Non-White Hispanic	513 (89.5)	542 (85.9)	562 (91.7)	2.2			-0.7 (-2.3, 1.0)
Education	Did not complete high school	2,048 (91.7)	1,738 (87.5)	1,428 (87.2)	-4.6	0.99	0.99	Ref
	High school or above	6,130 (95.0)	6,377 (92.5)	5,904 (91.8)	-3.2			1.8 (0.7, 2.9)
Marital status	Not married	3,978 (92.9)	3,774 (88.7)	3,560 (88.5)	-4.3	0.81	0.79	Ref
	Married	4,200 (95.4)	4,341 (93.9)	3,772 (93.0)	-2.4			3.0 (1.8, 4.1)
Living Situation	Lives alone	2,675 (93.4)	2,539 (88.7)	2,475 (89.5)	-4.0	0.81	0.87	-0.4 (-1.3, 0.6)
	Lives with others	5,503 (94.6)	5,576 (92.8)	4,857 (91.7)	-2.9			Ref
Primary insurance	TM	5,991 (94.1)	5,564 (90.6)	5,445 (90.5)	-3.6	0.35	0.29	-3.2 (-4.1, -2.3)
	MA	2,187 (94.5)	2,551 (93.5)	1,887 (92.4)	-2.1			Ref

© 2021 Ganguli I et al. *JAMA Network Open.*

Supplemental Insurance	Medicaid	933 (89.5)	979 (86.4)	985 (86.0)	-3.5	0.81	0.84	0.6 (-1.1, 2.4)
	Commercial	4,653 (95.8)	4,247 (93.4)	3,882 (93.2)	-2.6			3.7 (2.7, 4.8)
	None	2,592 (93.1)	2,889 (90.6)	2,465 (89.8)	-3.3			Ref
ACO participation	Enrolled in an ACO	--	874 (95.9)	1,658 (93.6)	--	--	--	
	Not enrolled in an ACO		7,241 (91.2)	5,674 (90.4)				
Rural urban	Metro	--	--	5,600 (8.5)	--	--	---	
	Micro			1,101 (87.7)				
	Rural			631 (91.1)				
Census areas (four)	Northeast	1,392 (94.2)	1,295 (92.6)	1,332 (94.5)	0.2	<.001	<.001	1.0 (-0.2, 2.1)
	Midwest	1,974 (95.0)	2,115 (92.9)	1,866 (91.7)	-3.4			Ref
	South	3,342 (95.2)	3,169 (91.0)	2,797 (90.3)	-4.9			-0.0 (-1.2, 1.2)
	West	1,457 (91.4)	1,532 (90.2)	1,337 (88.4)	-3.0			-3.1 (-4.6, -1.6)
Frailty quartiles	25%	1,989 (94.7)	2,073 (91.7)	1,890 (91.8)	-2.9	0.53	0.58	Ref
	50%	2,095 (94.9)	2,076 (92.4)	1,829 (91.6)	-3.3			-0.3 (-1.5, 1.0)

© 2021 Ganguli I et al. *JAMA Network Open.*

	75%	2,078 (94.3)	2,010 (91.3)	1,785 (89.9)	-4.4			-1.2 (-2.4, -0.1)
	100%	2,016 (92.8)	1,956 (90.7)	1,828 (90.3)	-2.5			-1.0 (-2.5, 0.6)
Number of chronic conditions	2	2,563 (93.8)	2,392 (90.1)	2,250 (90.1)	-3.7	0.74	0.75	Ref
	3	2,478 (94.1)	2,469 (91.9)	2,185 (91.1)	-3.0			1.5 (0.6, 2.5)
	4	1,715 (95.7)	1,752 (92.4)	1,559 (92.3)	-3.3			2.4 (1.2, 3.6)
	5+	1,422 (94.6)	1,502 (92.6)	1,338 (91.0)	-3.7			2.7 (1.4, 4.0)
Self-reported health	Excellent, very good or good, %	6,194 (94.8)	6,278 (92.3)	5,722 (91.4)	-3.3	0.44	0.45	1.2 (0.1, 2.2)
	Fair or poor, %	1,964 (93.2)	1,798 (88.9)	1,610 (89.5)	-3.7			Ref

SOURCE: Centers for Medicare & Medicaid Services, Medicare Current Beneficiary Survey, Survey File Data, 2010, 2013, 2016. All percentages are weighted. Income reported as don't know/refused was treated as a separate category. The multivariable model used pooled date from 2010, 2013, and 2016 and included all variables presented in the table other than ACO participation and rural-urban status (only asked in 2016). This multivariable model excluded 93 respondents for whom one or more responses were missing.

© 2021 Ganguli I et al. *JAMA Network Open.*

eTable 4. Trends and Patient and Area-Level Characteristics Associated With Reporting a Specialist as the Usual Clinician of Care, Additional Details

		2010	2013	2016	Difference 2010 – 2016	P value for trend test	P value for chi square, 2010 vs 2016	Adjusted marginal percentage point difference between subgroups
		No. (%) N = 8,116	No. (%) N = 8,005	No. (%) N = 7,158	% point			% point (95% CI) N = 23,204
**Overall**		**428 (5.3)**	**337 (4.4)**	**272 (4.1)**	-1.2	**<.001**	**<.001**	
Sex	Male	178 (5.5)	151 (4.7)	110 (4.2)	-1.3	0.55	0.48	Ref
	Female	250 (5.2)	186 (4.1)	162 (4.0)	-1.2			-0.7 (-1.5, 0.1)
Age	65-74	171 (5.3)	125 (4.1)	97 (4.1)	-1.3	0.78	0.81	Ref
	75-84	171 (5.1)	144 (4.6)	104 (3.8)	-1.3			0.0 (-0.6, 0.6)
	85+	86 (5.9)	68 (5.0)	71 (4.8)	-1.1			0.3 (-0.6, 1.2)
Income	<$15,000	93 (5.0)	68 (4.5)	50 (3.9)	-1.1	0.30	0.46	Ref
	$15,000-$29,999	101 (5.0)	96 (4.3)	76 (4.5)	-0.5			-0.5 (-1.4, 0.3)
	$30,000-$49,999	92 (5.4)	54 (3.3)	44 (3.1)	-2.2			-1.0 (-2.0, 0.1)
	$50,000+	91 (6.2)	81 (4.6)	102 (4.5)	-1.7			0.1 (-1.2, 1.3)
Race (RTI)	White	334 (5.1)	258 (4.2)	200 (3.7)	-1.4	0.40	0.41	Ref
	Black	41 (6.5)	31 (4.1)	30 (5.8)	-0.7			1.5 (0.2, 2.8)

© 2021 Ganguli I et al. *JAMA Network Open.*

	Asian/Native/Unknown/Other	14 (3.7)	17 (3.8)	12 (5.1)	1.4			0.6 (-1.2, 2.3)
	Non-White Hispanic	37 (7.2)	30 (6.5)	30 (5.6)	-1.7			3.8 (1.9, 5.7)
Education	Did not complete high school	91 (4.5)	61 (3.4)	46 (3.4)	-1.1	0.83	0.79	Ref
	High school or above	337 (5.6)	276 (4.6)	226 (4.3)	-1.3			1.4 (0.6, 2.3)
Marital status	Not married	220 (5.5)	189 (5.1)	146 (4.7)	-0.8	0.39	0.53	Ref
	Married	208 (5.2)	148 (3.8)	126 (3.6)	-1.6			-0.9 (-2.1, 0.2)
Living Situation	Lives alone	151 (5.7)	114 (4.9)	100 (4.9)	-0.8	0.89	0.90	0.4 (-0.6, 1.3)
	Lives with others	277 (5.2)	223 (4.1)	172 (3.8)	-1.4			Ref
Primary insurance	TM	353 (6.1)	268 (5.0)	226 (4.7)	-1.4	0.99	0.93	2.3 (1.6, 2.9)
	MA	75 (3.3)	69 (3.1)	46 (2.5)	-0.8			Ref
Supplemental Insurance	Medicaid	38 (4.1)	42 (3.9)	35 (3.6)	-0.6	0.55	0.57	-1.3 (-2.2, -0.3)
	Commercial	272 (6.1)	201 (5.0)	155 (4.4)	-1.7			0.4 (-0.5, 1.3)
	None	118 (4.4)	94 (3.5)	82 (3.8)	-0.6			Ref
ACO participation	Enrolled in an ACO	--	42 (5.1)	73 (5.0)	--	--	--	
	Not enrolled in an ACO		295 (4.3)	199 (3.9)				
Rural urban	Metro	--	--	234 (4.5)	--	--	--	
	Micro			28 (2.5)				

© 2021 Ganguli I et al. *JAMA Network Open.*

	Rural			10 (1.8)				
Census areas (four)	Northeast	86 (6.5)	87 (7.3)	75 (7.4)	0.9	0.23	0.28	3.6 (2.1, 5.2)
	Midwest	81 (4.3)	70 (3.4)	45 (2.8)	-1.5			Ref
	South	182 (5.5)	124 (4.1)	100 (3.4)	-2.2			0.7 (-0.2, 1.5)
	West	78 (5.0)	56 (3.4)	52 (3.8)	-1.2			0.4 (-0.5, 1.2)
Frailty quartiles	25%	79 (3.9)	79 (4.6)	64 (3.6)	-0.3	0.47	0.52	Ref
	50%	109 (5.6)	81 (3.7)	60 (4.0)	-1.6			0.8 (0.0, 1.6)
	75%	117 (5.6)	82 (4.6)	65 (4.5)	-1.1			1.1 (0.2, 2.1)
	100%	123 (6.4)	95 (4.6)	83 (4.6)	-1.9			1.6 (0.5, 2.8)
Number of chronic conditions	2	125 (5.2)	91 (4.1)	86 (4.5)	-0.7	0.46	0.43	Ref
	3	127 (5.0)	103 (4.6)	88 (4.3)	-0.8			-0.3 (-1.1, 0.6)
	4	89 (5.2)	79 (4.5)	50 (3.6)	-1.5			-0.8 (-1.8, 0.2)
	5+	87 (6.3)	64 (4.3)	48 (3.5)	-2.7			-0.7 (-1.7, 0.3)
Self-reported health	Excellent, very good or good, %	307 (4.9)	246 (4.2)	202 (4.0)	-0.9	0.99	0.99	-0.8 (-1.7, 0.1)
	Fair or poor, %	120 (6.7)	89 (5.1)	70 (4.5)	-2.2			Ref

SOURCE: Centers for Medicare & Medicaid Services, Medicare Current Beneficiary Survey, Survey File Data, 2010, 2013, 2016. All percentages are weighted. Income reported as don't know/refused was treated as a separate category. The multivariable model used pooled date from 2010, 2013, and 2016 and included all variables presented in the table other than ACO participation and rural-urban status (only asked in 2016). This multivariable model excluded 75 respondents for whom one or more responses were missing.

© 2021 Ganguli I et al. *JAMA Network Open.*

eFigure 2. Specialties of Usual Clinicians Among Respondents Reporting a Specialist in This Role, 2010-2016

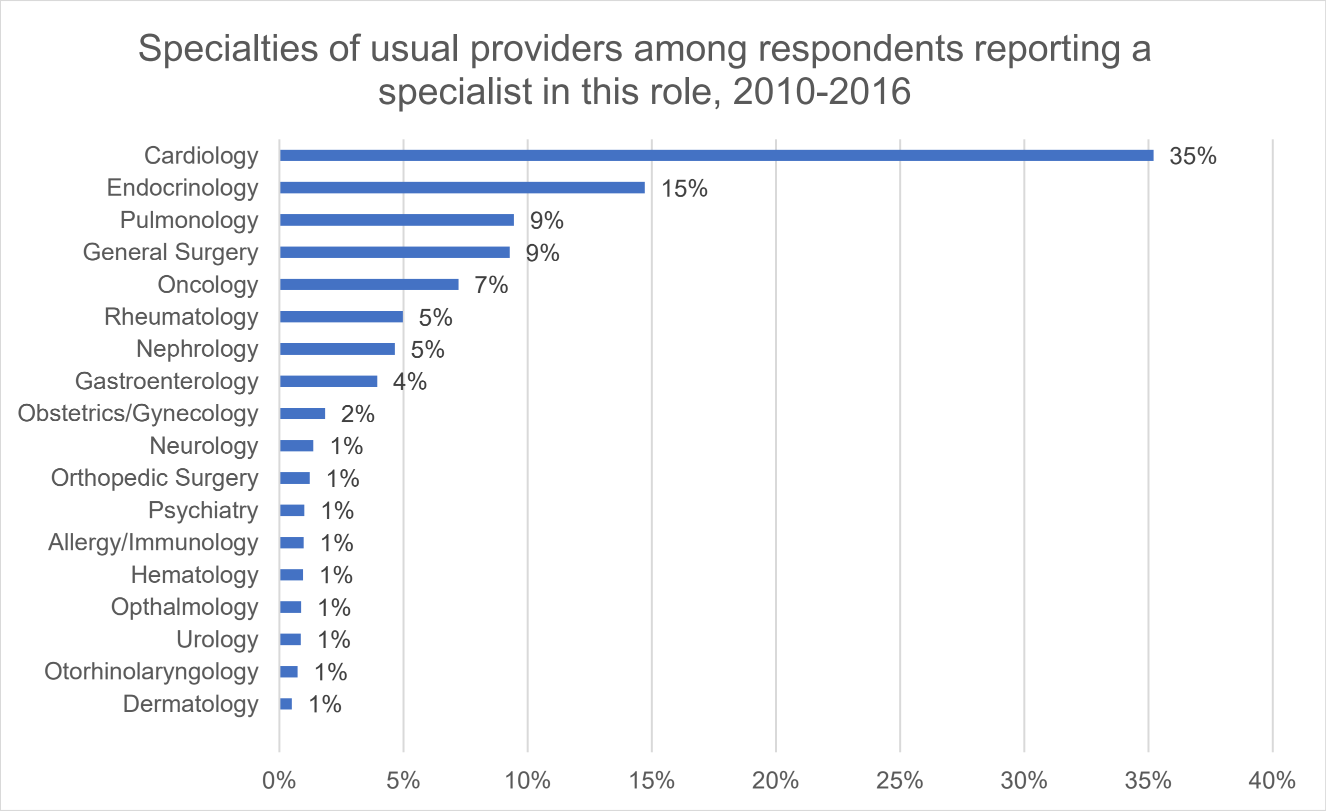

These numbers represented weighted percentages of responses pooled across the three years of analysis.

© 2021 Ganguli I et al. *JAMA Network Open.*
